# Recurrent Bacterial Meningitis in a Child with Mondini Dysplasia

**DOI:** 10.1155/2014/364657

**Published:** 2014-11-06

**Authors:** Eda Kepenekli-Kadayifci, Ayşe Karaaslan, Serkan Atıcı, Adem Binnetoğlu, Murat Sarı, Ahmet Soysal, Gülşen Altınkanat, Mustafa Bakır

**Affiliations:** ^1^Department of Pediatrics, Division of Pediatric Infectious Diseases, Pendik Training and Research Hospital, Marmara University Medical Faculty, Mimar Sinan Cad No. 41, Fevzi Cakmak Mah, Ust kaynarca-Pendik, 34899 Istanbul, Turkey; ^2^Department of Otorhinolaryngology, Pendik Training and Research Hospital, Marmara University Medical Faculty, Mimar Sinan Cad No. 41, Fevzi Cakmak Mah, Ust kaynarca-Pendik, 34899 Istanbul, Turkey; ^3^Marmara University Medical Faculty, Department of Medical Microbiology, 34899 Istanbul, Turkey

## Abstract

Mondini dysplasia, also known as Mondini malformation, is a developmental abnormality of the inner and middle ears that can cause hearing loss, cerebrospinal fluid (CSF) leakage, and recurrent bacterial meningitis (RBM), which is defined as two or more episodes of meningitis separated by a period of convalescence and the complete resolution of all signs and symptoms. An accurate diagnosis of the underlying pathology is crucial to prevent further episodes from occurring. Herein, we present a three-year-old boy with RBM and unilateral sensorineural hearing loss. During the evaluation to determine the etiology of the RBM, cystic malformation in the cochlea and vestibular dilatation consistent with Mondini dysplasia were detected via computerized tomography (CT) of the temporal bone.

## 1. Introduction

Recurrent bacterial meningitis (RBM) is defined as two or more episodes of meningitis that are separated by a period of convalescence and the complete resolution of all signs, symptoms, and laboratory findings [1–3]. The mortality rate for RBM episodes is lower than what is found with acute bacterial meningitis [2–5]. In previous studies mortality rates of RBM and acute bacterial meningitis are reported as 0–15% and %20–34, respectively [4, 5]. Lower mortality of RBM may be related to awareness of the patients who had experienced previous episodes of meningitis and their ability to recognize the symptoms of meningitis and seek medical help much earlier [2–4]. Nevertheless, identifying the underlying etiology of RBM is still crucial so that further episodes can be prevented. The predisposing factors for RBM can be broadly categorized into congenital and acquired conditions and then further divided into anatomic abnormalities, immunodeficiencies, and chronic parameningeal infections, with the most common cause being anatomic abnormalities [1–3]. A variety of cranial and spinal anatomic defects can facilitate the migration of microorganisms to the CSF spaces. Another frequent abnormality is congenital inner ear malformations, which is the leading cause of RBM episodes. When these are present, nasopharyngeal commensal microorganisms migrate from the nasopharynx to the middle ear via the Eustachian tube and travel on to the CSF spaces through the abnormal inner ear fistulous connections.

Mondini dysplasia, one of the more common congenital inner ear malformations, is thought to result from a developmental arrest of the interscalar septum in which the cochlea characteristically consists of one and a half turns instead of two and a half [2, 6, 7]. In addition, the vestibular structures and their associated neural elements may be similarly underdeveloped.

## 2. Case Report

A three-year-old boy presented to the emergency room with fever and decreased level of consciousness. Two days earlier, he had been admitted to another hospital with a presumed diagnosis of enteritis, and ceftriaxone therapy had been administered. A physical examination revealed fever, weakness, and nuchal rigidity. His medical history showed that he had been vaccinated four times with 13-valent pneumococcal conjugate vaccine (PCV-13) and that his father had been treated for meningitis when he was seven years old. A laboratory investigation revealed the following: a peripheric blood leukocyte count of 52.000/mm^3^, an absolute neutrophil count of 48.000/mm^3^, a platelet count of 413.000/mm^3^, and a C-reactive protein (CRP) level of 214 mg/L (normal range: 0–5 mg/L). Furthermore, the results of the CSF examination showed 200 polymorphonuclear leukocytes/mm^3^, an elevated protein concentration of 214 mg/dL, and a low glucose level of 1 mg/dL along with a simultaneous blood glucose level of 126 mg/dL. However, the CSF pyogenic culture and blood cultures remained sterile, and his cranial magnetic resonance imaging (MRI) was also normal. Ceftriaxone and vancomycin were administered for 14 days because his meningitis had only been partially treated with antibiotics before his admission. He was then discharged after full clinical and laboratory improvement.

Ten days after being discharged, the patient was readmitted with fever and neck stiffness, and he underwent another CSF examination that revealed 10 leukocytes/mm^3^, an elevated protein level of 209 mg/dL, and a low glucose level of 0 mg/dL. Additionally, his white blood cell (WBC) count was 27.200/mm^3^, and he had an absolute neutrophil count of 23.500/mm^3^ along with a CRP level of 91 mg/L. Moreover, the CSF polymerase chain reaction (PCR) test for* M. tuberculosis *was negative. Treatment with ceftriaxone and vancomycin was then restarted. The CSF culture revealed* Streptococcus pneumoniae *(serotype 19F) which was susceptible to both ceftriaxone and vancomycin, and a repeated lumbar puncture showed a cell count of 10 leukocytes/mm^3^, a CSF protein concentration of 41 mg/dL, a CSF glucose level of 35 mg/dL, and a simultaneous blood glucose level of 85 mg/dL, but the CSF pyogenic culture was sterile. Hence, the antibiotic therapy was given to the patient for four weeks.

**Figure 1:**
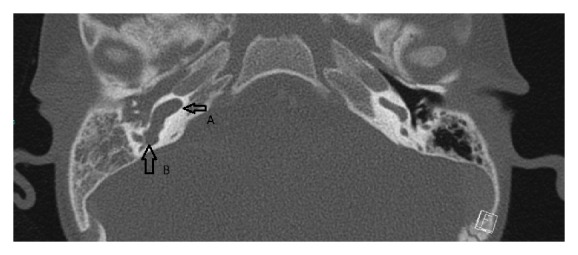
Computed tomography of the temporal bone showing the cystic malformation in the cochlea (A) and the vestibular dilatation (B).

**Figure 2:**
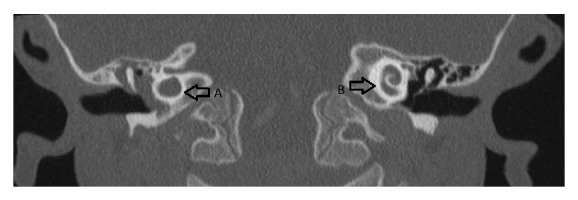
Computerized tomography of the temporal bone showing the dysmorphic cochlea in the right ear (A) and the normal cochlear structure in the left ear (B).

Cranial and temporal bone imagings were performed to determine the predisposing conditions for RBM, and temporal bone CT showed decreased aeration in the right middle ear and mastoid cells, a dysmorphic structure of the semicircular canals, vestibular dilatation, and the cystic appearance of the cochlea, all of which are consistent with Mondini dysplasia (Figures 1 and 2). Furthermore, an audiometric screening was consistent with severe sensorineural hearing loss in the right ear and normal hearing in the left ear.

Surgical repairment of the dysplastic inner ear was planned, but it could not be performed because of parental refusal.

## 3. Discussion

Bacterial meningitis is a life-threatening disease with high mortality and morbidity. Recurrent bacterial meningitis is rare, occurring in just 1% of all bacterial meningitis cases, and it poses a diagnostic challenge [6]. Early diagnosis of the underlying pathology is vital because it can prevent further episodes and improve the overall outcome of the patients [2, 3].

Anatomical abnormalities such as spinal defects or abnormalities in the anterior fossa or temporal bone represent the most common cause of recurrent meningitis [1, 8]. Mondini dysplasia is the most frequent anomaly associated with CSF leaks originating in the temporal bone in children. These leaks can cause recurrent meningitis, hearing impairment, otorrhea, or rhinorrhea [6, 9]. Examples of temporal bone anomalies that can lead to RBM are Mondini dysplasia, stapedial anomalies, Klippel-Feil syndrome, Pendred syndrome, petromastoid fistulae, a widened cochlear aqueduct, and Hyrtl's fissure [1]. Mondini dysplasia is a developmental anomaly of the middle ear, cochlea, and vestibule. An abnormal connection between the subarachnoid space and middle ear may result in RBM, which is also associated with hearing deficits [6]. Our patient did not have obvious otorrhea, rhinorrhea, or hearing loss complaints, but his audiometric screening was consistent with severe sensorineural hearing loss in the right ear.

Communication between the middle ear and CSF spaces may result in bacterial translocation, with the most common infecting organism being* S. Pneumoniae* [1–8, 10]. In our patient, no microorganism was detected in the first meningitis episode, but this may have been related to the previous ceftriaxone therapy before his admission. At the second meningitis episode, the CSF culture revealed* S. Pneumoniae, *which is the infectious agent in more than 50% of patients with RBM [1–8].* Neisseria meningitidis* is the second most common agent, but it is associated more with immunological abnormalities [1–3].

In RBM, imaging studies should include CT of the cranium, paranasal sinuses, and temporal bones, and multiple coronal views must be obtained because small defects can be missed in axial views [1, 11, 12]. In our patient, Mondini dysplasia was suspected because of the RBM and unilateral hearing loss. This diagnosis was confirmed via the temporal bone CT that showed cystic malformation in the cochlea and vestibular dilatation in the right ear but normal cochlear structure in the left ear (Figures 1 and 2).

Treatment options in these cases include the surgical repair of the defect to prevent recurrent meningitis, amplification aids for those with residual hearing, and cochlear implants [13, 14]. When developmental or traumatic anatomical defects are present, the pediatrician needs to consult with a neurosurgeon or otolaryngologist to evaluate the necessity for surgical repair [11]. If needed, surgical methods such as transmastoid approach, middle fossa craniotomy, and combined approaches to repair of spontaneous temporal bone CSF leak are highly successful [13, 14].

In conclusion, we recommend CT of the temporal bones in patients with recurrent meningitis and hearing loss to assess whether or not Mondini dysplasia is present.
